# Urinary Complications and Injuries Related to Vaginal Mesh Surgery for Pelvic Organ Prolapse: Prevention and Management

**DOI:** 10.3390/jcm15197535

**Published:** 2026-09-28

**Authors:** Aleksandra Piątek, Gabriela Kołodyńska, Krystian Kaczmarek, Mateusz Maślanka, Waldemar Andrzejewski, Maciej Zalewski

**Affiliations:** 1 Department of Gynecology, Independent Public Health Care Center of the Ministry of the Interior and Administration in Wrocław, 50-233 Wrocław, Poland; 2 Department of Physiotherapy, Wroclaw University of Health and Sport Sciences, 51-612 Wrocław, Poland; 3 Department of Urology and Urological Oncology, Pomeranian Medical University, 70-204 Szczecin, Poland; 4 Department of Descriptive and Clinical Anatomy, Medical University of Warsaw, 02-091 Warsaw, Poland

**Keywords:** pelvic organ prolapse, vaginal mesh, postoperative urinary retention, ureteral obstruction, bladder injury, urinary tract complications

## Abstract

**Background/Objectives:** Pelvic organ prolapse affects up to one in five postmenopausal women. Although transvaginal mesh procedures are restricted to selected cases, they may be followed by transient postoperative urinary retention (POUR) and, rarely, serious urinary tract complications. This study assessed the frequency and risk factors for urinary complications after vaginal mesh prolapse repair and described strategies for their prevention, early detection, and management. **Methods:** This retrospective, single-center observational study with nested case series included 517 postmenopausal women aged 48–90 years who underwent anterior and apical prolapse repair using the ultralight polypropylene InGYNious anterior system between January 2021 and March 2026. The primary outcome was POUR. Secondary outcomes included urinary tract infection, bladder or ureteral injury, hydronephrosis, oliguria, acute kidney injury, and the need for urological intervention. **Results:** POUR occurred in 128 of 517 patients (24.8%) within 24 h after catheter removal; 45 of these women (35.2%) required catheterization and retention persisted beyond the first postoperative day in 101 (78.9%). Seven urinary tract injuries were recorded, including four bladder injuries and three cases of ureteral injury or kinking. Four severe postoperative cases were analyzed in detail. Concomitant posterior colporrhaphy was independently associated with POUR (OR = 1.97, 95% CI 1.31–2.97; 31.1% vs. 18.6%) and with any urinary tract complication (OR = 1.52, 95% CI 1.03–2.25). Age, parity, and body mass index were not significantly associated with these outcomes. **Conclusions:** Careful preoperative evaluation, appropriate surgical training, avoidance of excessive tissue traction, and prompt assessment of postoperative oliguria are essential. Patients should be informed about the possibility of transient urinary retention and the potential need for short-term catheterization.

## 1. Introduction

Pelvic organ prolapse (POP) affects up to one in five postmenopausal women. Sacrocolpopexy or sacrocervicopexy is widely used for apical prolapse repair; however, vaginal prolapse surgery, including transvaginal mesh repair, may be considered in selected patients, particularly older or obese women and those with a history of abdominal surgery. Vaginal mesh procedures may be associated with dyspareunia, mesh exposure, or erosion and therefore require careful patient selection.

According to the European Urogynaecological Association (EUGA), vaginal mesh surgery may be considered in strictly selected cases, including recurrent prolapse, advanced apical defects (POP-Q stage III or IV), and patients at high risk of recurrence when native-tissue repair is considered insufficient. High-risk conditions include disorders associated with increased intra-abdominal pressure, such as obesity and chronic respiratory disease, and connective-tissue disorders [1].

In addition to appropriate clinical indications, patients must be informed about mesh-specific complications. Mesh procedures should be performed by urogynecologists with dedicated training in both implantation and the management of mesh-related complications [1]. Although serious urinary complications are uncommon, their consequences may be severe.

Postoperative urinary retention (POUR), characterized by impaired bladder emptying and an increased post-void residual volume, may occur in approximately 30% of patients after pelvic organ prolapse surgery [2,3].

Severe urological complications may result from laceration, ligation, or thermal injury to structures located close to the uterus, particularly the bladder and ureters [4]. Gynecologic surgery accounts for a substantial proportion of iatrogenic ureteral injuries. Bladder injury has been reported in approximately 0.24% of benign and up to 3.7% of oncologic gynecologic procedures, whereas ureteral injury rates range from approximately 0.1% to 1.1% [4].

The aim of this study was to evaluate the frequency and potential risk factors for urinary complications after vaginal mesh repair of anterior and apical prolapse and to describe the prevention, detection, and management of severe complications based on cases treated in our Department of Gynecology.

## 2. Materials and Methods

### 2.1. Study Design and Participants

This retrospective, single-center with nested case series observational study included a case series of severe urinary complications. We assessed urinary symptoms and complications in women who underwent surgical repair of anterior and apical prolapse between January 2021 and March 2026. The study included 517 postmenopausal women aged 48–90 years. All patients had an anterior vaginal wall defect with a concomitant apical defect classified as POP-Q stage III C or IV C, with or without a posterior vaginal wall defect.

The inclusion criteria were concurrent anterior and apical defects; POP-Q stage III or IV in at least one of these two compartments; female sex; age ≥ 18 years; absence of current sexual activity; refusal of pessary treatment or ineffective conservative management; and informed consent for surgery. The exclusion criteria were age < 18 years, current or planned regular sexual activity, current genitourinary infection, and lack of consent for surgery.

Anterior and/or apical prolapse is managed at the study center by a transabdominal approach (sacrocolpopexy, lateral suspension, paravaginal repair), by native-tissue vaginal repair, or by transvaginal mesh. Allocation to a surgical route was not random and followed institutional policy with the EUGA position statement [1]. The InGYNious anterior system (A.M.I. GmBH, Feldkirch, Austria) was reserved for women with recurrent prolapse or a high risk of recurrence, in whom native-tissue repair was considered insufficient and in whom an abdominal laparoscopic approach was undesirable or contraindicated, most often because of advanced age, obesity or comorbidities precluding general anesthesia, prolonged pneumoperitoneum or Trendelenburg positioning. During the study period, 517 women underwent cervical or vaginal vault suspension with the InGYNious anterior system at the study center, all with complete perioperative records.

Women who were sexually active or intended to resume regular sexual activity were not offered transvaginal mesh as a method of choice, in line with the European Urogyneacological Association position statement, because of the risk of dyspareunia or mesh exposure [1]. Such women were counseled toward abdominal or native-tissue repair.

Because the study was retrospective, the requirement for informed consent for retrospective data analysis was waived by the Bioethics Committee. All data were analyzed in a coded form.

### 2.2. Variable Definitions

Demographic, clinical, urogynecological, and perioperative variables were extracted retrospectively from the patients’ medical records. For statistical analyses, age was categorized as <60 or ≥60 years, and body mass index (BMI) as <25 or ≥25 kg/m^2^. Smoking status was recorded as a binary variable (no/yes).

Obstetric history included the number of vaginal deliveries and Caesarean sections. Vaginal delivery was categorized as 0–1 versus ≥2 deliveries, and Caesarean section as 0 versus ≥1. Time since the last menstrual period was categorized as ≤10 versus >10 years. Previous hysterectomy and previous surgery for pelvic organ prolapse (POP) were recorded as binary variables (no/yes). Preoperative urinary and pelvic floor symptoms were obtained from the medical records. Stress urinary incontinence (SUI), overactive bladder (OAB) with or without urgency urinary incontinence (UUI), increased post-void residual urine volume, and obstructed defecation were recorded as present or absent according to the preoperative clinical assessment documented in the medical record. SUI was recorded when a patient reported regular urine leakage during coughing, sneezing, laughing, or exercise. UUI/OAB was recorded when an urgency component was present, with or without leakage. Obstructed defecation was recognized when patients reported straining with or without a sensation of incomplete evacuation, or the need for digital assistance. Postoperative hematoma was defined as any localized collection of blood or clot outside the vessel, deep or superficial to the mesh or adjacent to the ischial spine, visualized on ultrasound performed before discharge. It was recorded irrespective of symptoms and of the need for drainage. Surgical revision of hematoma was recorded separately.

Relevant comorbidities, including diabetes mellitus, hypertension, dementia or previous stroke, depression, and anemia, were recorded as present or absent based on the documented medical history. Pelvic organ prolapse was assessed using the Pelvic Organ Prolapse Quantification (POP-Q) system, with grading from 0 to 4. Surgical procedures were categorized as anterior/apical mesh repair alone or anterior/apical mesh repair combined with concomitant posterior colporrhaphy.

### 2.3. Surgical Procedure and Perioperative Care

All patients had medical indications for cervical or vaginal vault suspension using the ultralight polypropylene InGYNious anterior system (A.M.I. Agency for Medical Innovations GmbH, Feldkirch, Austria). Preoperative and postoperative care followed the standards used in the study center.

Patients were instructed to apply local estrogen twice weekly for 4 weeks before surgery and to continue treatment after the procedure. When estrogen was contraindicated or poorly tolerated, hyaluronic acid or local prasterone was recommended.

Follow-up visits were scheduled within 6 weeks after surgery, at 6–12 months, and annually thereafter to facilitate early detection and treatment of complications.

Patients were admitted on the day before surgery. An enema was used only at the patient’s request. Perioperative antibiotic prophylaxis consisted of cefazolin 2 g in patients weighing < 130 kg and cefazolin 3 g in patients weighing ≥ 130 kg. In patients with allergy, clindamycin 900 mg was administered. The perineum and adjacent thighs were disinfected, followed by vaginal disinfection and isolation of the anus.

### 2.4. Mesh Placement Technique

The patient was placed in the dorsal lithotomy position under regional or general anesthesia. The InGYNious anterior mesh has a symmetrical six-arm trapezoidal design, with two elongated arms intended for bilateral fixation to the sacrospinous ligaments. The panel uses six-point suture fixation to create a hammock supporting the bladder and the vaginal vault or cervix (Figure 1). The mesh was fixed using nonabsorbable sutures placed with the dedicated i-Stich fixation system.

The surgical technique comprised the following steps:

- Submucosal hydrodissection of the anterior vaginal wall with 60–100 mL of 0.9% saline to facilitate dissection;
- Incision of the anterior vaginal wall from approximately 3 cm proximal to the urethral meatus to 2–3 cm proximal to the vaginal vault;
- Blunt and/or sharp dissection of the vaginal epithelium from the underlying bladder and preparation toward the ischial spine to identify the sacrospinous ligament (SSL) and arcus tendineus fasciae pelvis (ATFP);
- Preparation toward the pubic bone to expose the anterior part of the ATFP;
- Placement of six sutures with the i-Stich system, three on each side: DeLancey level 1 support in the SSL approximately 3 cm caudal to the ischial spine; level 2 support in the ATFP approximately 2 cm above the ischial spine or in the obturator fascia with the underlying obturator muscle and membrane; and level 3 support in the ATFP behind the pubic ramus approximately 4 cm lateral to the pubic symphysis;
- Fixation of the inferior mesh panel with three to five nonabsorbable sutures to the anterior aspect of the cervix or vaginal vault after separation from the bladder;
- Passage of the sutures through six corresponding points of the mesh outside the body;
- Repositioning of the cervix and bladder with the mesh to the desired position;
- Advancement of the sutures into the paravesical space, tension-free knotting, and short trimming of the suture ends;
- Limited trimming of redundant vaginal tissue and closure of the vaginal incision with a running or interrupted suture;
- Placement of moist vaginal packing.

Vaginal packing and the urinary catheter were usually removed on the morning after surgery. Post-void residual urine volume (PVR) was assessed by transabdominal or transvaginal ultrasonography after spontaneous voiding, from 4 to 6 h after catheter removal and every subsequent day during hospitalization. PVR was estimated with the volume ellipsoid formula.

### 2.5. Outcomes

The primary outcome was POUR, defined as impaired spontaneous voiding and/or an increased post-void residual urine volume after catheter removal that required observation, pharmacological treatment, intermittent catheterization, or reinsertion of an indwelling catheter. Asymptomatic women with a PVR of 150 to 500 mL were managed expectantly, with mobilization, repeated measurement, with or without pharmacotherapy. Reinsertion of an indwelling catheter was performed when the patient was unable to void, or when PVR exceeded 500 mL with accompanying lower abdominal pain. Persistent POUR was defined as retention meeting these criteria on more than one postoperative day.

The composite secondary outcome was defined as the occurrence of at least one of the following: POUR, symptomatic urinary tract infection, bladder or ureteral injury, hydronephrosis, oliguria, acute kidney injury, and the need for urological intervention.

Symptomatic urinary tract infection was defined as a positive urine culture (at least 10^5^ colony-forming units/mL of a single uropathogen) with clinical symptoms. Bladder injury was defined as a full-thickness cystotomy identified intraoperatively or at cystoscopy. Ureteral injury or kinking was defined as postoperative uni- or bilateral hydronephrosis with an impaired or absent ureteral jet, confirmed by cystoscopy, abdominal ultrasonography or/and computed tomography, or by the need for ureteral stenting, nephrostomy or mesh revision.

Intraoperative cystoscopy was not performed routinely; it was performed when bladder injury was suspected and in all patients with early postoperative oliguria or suspected ureteral compromise.

### 2.6. Statistical Analysis

Baseline demographic and clinical characteristics were compared between patients with and without urinary tract complications. Normality was assessed using the Shapiro–Wilk test. Continuous variables are presented as medians with interquartile ranges (IQRs) and were compared using the Mann–Whitney U test because of non-normal distributions. Categorical variables are presented as absolute numbers and percentages and were compared using the chi-squared test or Fisher’s exact test, as appropriate. Variables included age, body mass index (BMI), smoking status, obstetric and gynecologic history, urinary symptoms, comorbidities, POP-Q stage, and type of surgical procedure.

Univariable and multivariable logistic regression analyses were performed to identify potential factors associated with overall urinary tract complications and POUR. Odds ratios (ORs) with 95% confidence intervals (CIs) were calculated. Variables of clinical relevance and/or with *p* < 0.10 in univariable analysis were considered for the multivariable model. Forward stepwise logistic regression was used to identify independent predictors. A two-sided *p*-value < 0.05 was considered statistically significant. Because of the retrospective design, no a priori sample-size calculation was used to determine recruitment, and all consecutive eligible patients treated during the predefined study period were included. The adequacy of the available sample for multivariable logistic regression was assessed by considering the number of observed outcome events relative to the number of candidate predictor parameters entering the multivariable model. An event-to-parameter ratio of at least 10 was considered adequate to limit the risk of model overfitting. Analyses were performed using Statistica 13 (TIBCO Software Inc., Palo Alto, CA, USA).

## 3. Results

### 3.1. Cohort Characteristics and Postoperative Urinary Retention

Baseline characteristics of the 517 patients according to the presence or absence of urinary tract complications are presented in Table 1. The 149 recorded events occurred in 141 women, 8 of whom had two concurrent complications.

No significant differences were observed for age, smoking, obstetric history, previous prolapse surgery, urinary symptoms, or most comorbidities. The type of procedure differed between groups (*p* = 0.043). Previous prolapse surgery had been performed in 61 women (11.8%), of whom 27 had more than one procedure. Anterior colporrhaphy had been performed in 34 women (14 as an isolated compartment repair), posterior colporrhaphy in 23 (including 4 as the sole procedure), an apical suspension in 22 patients (including sacrospinous ligament fixation with or without mesh, abdominal cervical suspension, or hysteropexy), a Manchester procedure in 2, and a paravaginal repair in 1. In 7 women the exact type of previous repair was not recorded.

Follow-up data were available for 481 of 517 patients (93.0%) within 3 months after surgery. Follow-up completeness decreased over time, with 237 patients (45.8%) evaluated at 6–12 months, 94 (18.2%) at 2 years, 33 (6.4%) at 3 years, 12 (2.3%) at 4 years, and 3 (0.6%) at 5 years. As follow-up was recorded in predefined time intervals rather than as exact dates of individual visits, a precise median follow-up duration could not be calculated.

POUR occurred in 128 patients (24.8%) within 24 h after catheter removal. Among patients with POUR, 83 (64.8%) were managed conservatively or pharmacologically, whereas 45 (35.2%) required catheterization because of impaired voiding. In 101 patients (19.5% of the total cohort), urinary retention persisted for more than 1 day, this corresponded to 78.9% of the 12 8 women with POUR. POUR occurred in 49 of 263 women (18.6%) after anterior and apical repair alone and in 79 of 254 (31.1%) when posterior colporrhaphy was added. The corresponding figures for any urinary complication were 23.2% and 31.5%. The frequencies of urinary complications are summarized in Table 2.

Ultrasonographically detected paravesical or paravaginal hematoma was recorded in 79 women (15.3%), of whom 18 cases (22.8% of hematomas) underwent surgical evacuation. Hematomas were not associated with POUR (*p* = 0.490). Mean operating time was 57.55 min in patients with retention on the first postoperative day and 60.58 min in patients without retention; the difference was not significant. Similarly, operating time did not differ between patients with and without retention on subsequent days (57.81 vs. 60.35 min; *p* = 0.255).

In univariable logistic regression, age, BMI, smoking, obstetric history, urinary symptoms, comorbidities, and hematoma were not associated with POUR. Advanced posterior POP-Q stage and the combined anterior/apical repair with concomitant posterior colporrhaphy were associated with higher odds of POUR (Table 3).

Among the 22 potential predictors evaluated in univariable analyses, two met the predefined statistical criterion of *p* < 0.10 for consideration in the multivariable analysis. In the subsequent multivariable analysis, concomitant posterior colporrhaphy remained independently associated with POUR (OR = 1.97, 95% CI 1.31–2.97; *p* = 0.001; Table 4) and with any urinary tract complication (OR = 1.52, 95% CI 1.03–2.25; *p* = 0.035; Table 5).

### 3.2. Severe Upper Urinary Tract Complications: Case Series

Four patients developed severe postoperative urinary complications: two had acute kidney injury with suspected ureteral obstruction, one had hydronephrosis associated with ureteral kinking, and one had transient oliguria related to postoperative anemia. All patients had received preoperative counseling and had provided informed consent for surgery. These cases were presented to illustrate the description, differential diagnosis and management of severe upper urinary tract complications, which are too infrequent for quantitative analysis, but define the severe end of the spectrum of urinary tract risks associated with mesh implementation.

#### 3.2.1. Patient 1

A 48-year-old woman with total vaginal prolapse and preoperative urinary retention had schizophrenia, chronic kidney disease, and a history of one vaginal delivery. On the first postoperative day, urine output was 300 mL/24 h, and serum creatinine increased from 1.13 to 4.34 mg/dL within 36 h. Contrast-enhanced computed tomography was contraindicated because of the low eGFR. Ultrasonography showed marked hydronephrotic dilatation of the collecting systems. The right ureter could not be clearly visualized; the left ureter was dilated to 9 mm along its course beyond the crossing of the iliac vessels. Compression of the distal ureters was suspected, and bilateral double-J ureteral stents were inserted.

#### 3.2.2. Patient 2

A 77-year-old woman without major chronic disease, with a history of one vaginal delivery, was referred with suspected left ureteral kinking. Two weeks after mesh implantation, she developed left lumbar pain, and ultrasonography showed hydronephrosis despite normal laboratory findings. Because retrograde double-J stent placement was unsuccessful, a nephrostomy was performed. Partial removal of the two left mesh arms fixed to the ATFP was subsequently undertaken. Cystoscopy initially showed neither a ureteral jet nor the possibility of retrograde stent placement. Two weeks later, the nephrostomy was inadvertently removed; however, repeat ultrasonography showed no recurrent hydronephrosis, and a spontaneous left ureteral jet was visible on transvaginal ultrasonography.

#### 3.2.3. Patient 3

A 69-year-old woman passed only 150 mL of urine on the first postoperative day. Initially, acute kidney injury of presumed prerenal origin was considered because of her history of lymphoma, and hydration and diuretic treatment were initiated. Abdominal ultrasonography showed hydronephrosis but did not adequately visualize the distal ureters. On the second day, progressive dilatation of the pelvicalyceal systems and ureters was observed, and serum creatinine increased from 0.91 to 6.06 mg/dL, suggesting postrenal acute kidney injury. Total mesh removal was performed within 48 h. Intraoperative cystoscopy confirmed restoration of bilateral ureteral jets. Within 1 week, ultrasonographic abnormalities regressed and serum creatinine decreased to approximately 1 mg/dL.

#### 3.2.4. Patient 4

A 77-year-old woman with rheumatoid arthritis, diabetes, hypertension, bronchiectasis, anemia, mild dementia, previous breast cancer surgery, early menopause at 40 years of age, and one vaginal delivery underwent surgery for total vaginal prolapse with a preoperative residual urine volume of 300 mL and bilateral hydronephrosis. Estimated blood loss was 800–1000 mL, and hemoglobin decreased from 10.0 to 7.9 g/dL. During the first 24 h, only 200 mL of urine was collected through the catheter. Ultrasonography showed improvement of hydronephrosis and a 60 × 50 mm hematoma, while creatinine and electrolyte concentrations remained normal. After transfusion of two units of red blood cells, the patient’s condition improved and 1200 mL of urine was collected within 2 h, indicating transient oliguria related to postoperative anemia and hypoperfusion.

## 4. Discussion

### 4.1. Diagnostic and Management Considerations

Postoperative oliguria after vaginal mesh surgery for POP requires rapid and comprehensive evaluation to distinguish prerenal, intrinsic renal, and postrenal causes [4,5]. Initial assessment should include fluid balance, estimated blood loss, and hemodynamic status, because hypotension and postoperative anemia may cause transient renal hypoperfusion. Laboratory investigations should include a complete blood count, serum creatinine, blood urea nitrogen, electrolytes, and eGFR to facilitate early detection of acute kidney injury [4,6].

Urinary tract ultrasonography is essential for the assessment of hydronephrosis and post-void residual urine volume. When bladder outlet obstruction or ureteral kinking is suspected, urgent urological consultation should be obtained. Further assessment may include cystoscopy, retrograde ureteral catheterization, or computed tomography when clinically feasible [5,7]. Contrast-enhanced CT urography is generally limited to patients with adequate renal function.

If retrograde ureteral catheterization is not possible because of severe kinking or complete obstruction, temporary percutaneous nephrostomy should be considered to decompress the upper urinary tract and preserve renal function [5,7]. Nephrostomy also enables antegrade pyelography to determine the level of obstruction or injury and to identify urinary extravasation. Delayed intervention may increase the risk of irreversible renal damage and ureteral devascularization [7].

### 4.2. Bladder Injury

Bladder injury is uncommon during gynecologic surgery. In the four bladder injuries observed in this cohort, the lesions were small and were managed with an indwelling catheter for 7 days without additional antibiotic therapy. No inflammatory complications or other sequelae were observed. Bladder injury may nevertheless lead to complications such as vesicovaginal fistula. A systematic review found no clear association between catheterization duration and adverse outcomes after iatrogenic bladder injury [8].

Standardized recommendations for bladder repair remain limited. Polyglactin and poliglecaprone sutures are commonly used because they are absorbable and cause limited tissue reaction. Injuries are typically closed using an unlocked running suture in one or two layers or with interrupted sutures [9].

### 4.3. Ureteral Kinking and Obstruction

Mesh implantation may cause serious urinary tract complications. In the three patients with hydronephrosis or postrenal impairment, ureteral kinking was considered the most likely mechanism, although it could not be confirmed directly in every case. In Patient 3 this interpretation is supported by the restoration of bilateral ureteral jets at cystoscopy and by the rapid fall in serum creatinine after mesh removal, whereas in Patients 1 and 2 it remains a clinical inference based on imaging and response to decompression. In Patient 2, fixation near the ischial spine or the ATFP may have altered the distal ureteral anatomy. Long-standing total prolapse may substantially distort anatomic relationships. A second potential mechanism is excessive tissue retraction during prolapse reduction (Figure 2).

Similar findings have been described with the four-corner UpHold implant, in which the cervix and pubocervical fascia are suspended to the sacrospinous ligaments. Traction on the lateral arms repositions the cervix and bladder neck deeper in the pelvis and may change the angle of the ureteral orifice, resulting in ureteral kinking or obstruction [10].

Strong repositioning with the InGYNious system may similarly distort the ureterovesical junction. In Patient 3, bilateral ureteral obstruction may have resulted from excessive posterior relocation, although this explanation is hypothetical and is offered to prompt further studies rather than to establish causation. Because this complication is rare, individual anatomic abnormalities at the ureterovesical junction may also contribute.

The ureters normally pass approximately 1.5 cm lateral to the cervix. Cadaveric studies have reported a minimum distance of 0.5–1.5 cm between the ureter at the uterine artery crossing and a mesh arm anchored to the sacrospinous ligament; in fresh cadavers, the distance may be as little as 5 mm [11]. Although direct injury may be unlikely when sutures are placed in fascia rather than near the bladder neck [10], routine cystoscopy after vaginal mesh surgery has been proposed as a method of detecting obstruction or injury [10].

Ureteral kinking can present with renal colic and ultrasonographically confirmed hydronephrosis. Reported management after UpHold implantation has included double-J stenting and removal of the affected mesh arm. Even after radiologic resolution, delayed ureteral reimplantation may be required because chronic kinking can cause distal ureteral devascularization [7]. Ureteral obstruction has also been reported after sacrospinous fixation with an Avaulta Plus mesh, requiring ureteral transection and reimplantation [12].

The distal ureter receives branches from the bladder and internal iliac arterial systems and may be vulnerable to devascularization during dissection or chronic traction [7]. Approximately half of iatrogenic ureteral injuries are recognized intraoperatively. In one pilot study of 48 paravaginal mesh repairs, one bladder injury and two cases of ureteral kinking were reported [13]. In a cadaveric study, one of ten sacrospinous mesh fixations contacted and placed traction on the ureter [14].

### 4.4. Postoperative Urinary Retention

The reported risk of POUR after vaginal prolapse surgery without an anti-incontinence procedure is approximately 29%. Reported risk factors include older age, longer operating time, age ≥ 60 years, menopausal status, and greater blood loss [2,15]. Longer procedures may reflect technically difficult surgery, adhesions after previous prolapse operations, or greater blood loss and postoperative physiologic stress.

Rates of POUR are difficult to compare across series, because the International Continence Society (ICS) defines retention in clinical terms, and specifies no volume threshold [16]. We therefore applied our own definition of 150 mL in order to standardise management for all the patients.

POUR usually resolves without additional intervention and rarely persists beyond 4 weeks. Mesh stretching, incision, or resection should be considered when retention does not resolve spontaneously [3]. Additional reported risk factors include a preoperative post-void residual volume > 200 mL, severe cystocele, anterior repair, and concomitant hysterectomy [2,15].

In the present study, concomitant posterior colporrhaphy was the principal independent factor associated with both POUR and any urinary tract complication. This association is derived from an observational cohort in which the choice of procedure was determined by the extent of prolapse. We cannot exclude confounding by the severity of multicompartment prolapse; the association should not be interpreted as a causal effect of the posterior repair itself.

Several mechanisms may explain the association between concomitant posterior repair and transient voiding dysfunction, which is likely to be multifactorial. First, posterior colporrhaphy adds a second dissection field and a perineal wound. Pain caused by surgical trauma to the perineum and levator ani may inhibit voiding through a reflex caused by increased pelvic floor and external sphincter tone [17].

Second, plication of the rectovaginal fascia and narrowing of the levator hiatus alter the axis and the mobility of the vagina and, by local edema, may increase periurethral resistance.

Third, two-compartment surgery prolongs the operation, causes more tissue trauma, and delays mobilization. Together with greater blood loss and postoperative anemia, these factors have been reported to contribute to POUR after prolapse operation [2,15].

No association was found between POUR and postoperative hematoma, although drainage may relieve urethral compression in selected cases.

A neuropelveological explanation may also be considered, as prolonged POUR has not been recorded after abdominal route, such as sacropexy [18].

### 4.5. Limitations

This study has several limitations. First, it was retrospective and conducted at a single center. Second, the proposed mechanisms of ureteral obstruction and renal failure are based on the most plausible clinical interpretation rather than direct intraoperative confirmation in every case. Third, postoperative voiding dysfunction is multifactorial, and the available variables may not capture all contributing factors. Fourth, the cohort is highly selected: all women were postmenopausal and not sexually active, and all were treated with a specific mesh system, in a center certified for mesh training. The findings should therefore not be extrapolated to other mesh systems or native-tissue repair or different groups of patients. Finally, although early postoperative follow-up was available for the majority of patients, follow-up completeness decreased substantially over time, limiting the systematic assessment of long-term urinary tract outcomes.

## 5. Conclusions

Patients considered for vaginal mesh prolapse repair should undergo careful urogynecologic evaluation, including POP-Q staging, measurement of post-void residual urine volume, renal ultrasonography, and appropriate laboratory testing, including complete blood count, glucose, electrolytes, and serum creatinine.

Vaginal mesh surgery should be performed by appropriately trained surgeons with vigilance for rare but serious urinary tract complications. Patients should be informed about transient POUR and the possible need for short-term self-catheterization or catheter reinsertion.

Excessive posterior traction and overcorrection may distort the ureterovesical junction in susceptible patients, although this mechanism remains a hypothesis derived from a small number of observed cases rather than a demonstrated causal pathway. Long-standing prolapse-related anatomic distortion and incorrect anchor placement may further increase the risk of ureteral kinking. Prompt diagnostic evaluation and relief of obstruction are critical to preserve renal function.

These conclusions are drawn from a single-center exploratory retrospective cohort treated with one mesh system and apply primarily to comparable patients and settings. The frequency estimates and the proposed mechanisms require confirmation in prospective, multicenter studies with systematic long-term follow-up.

## Figures and Tables

**Figure 1:**
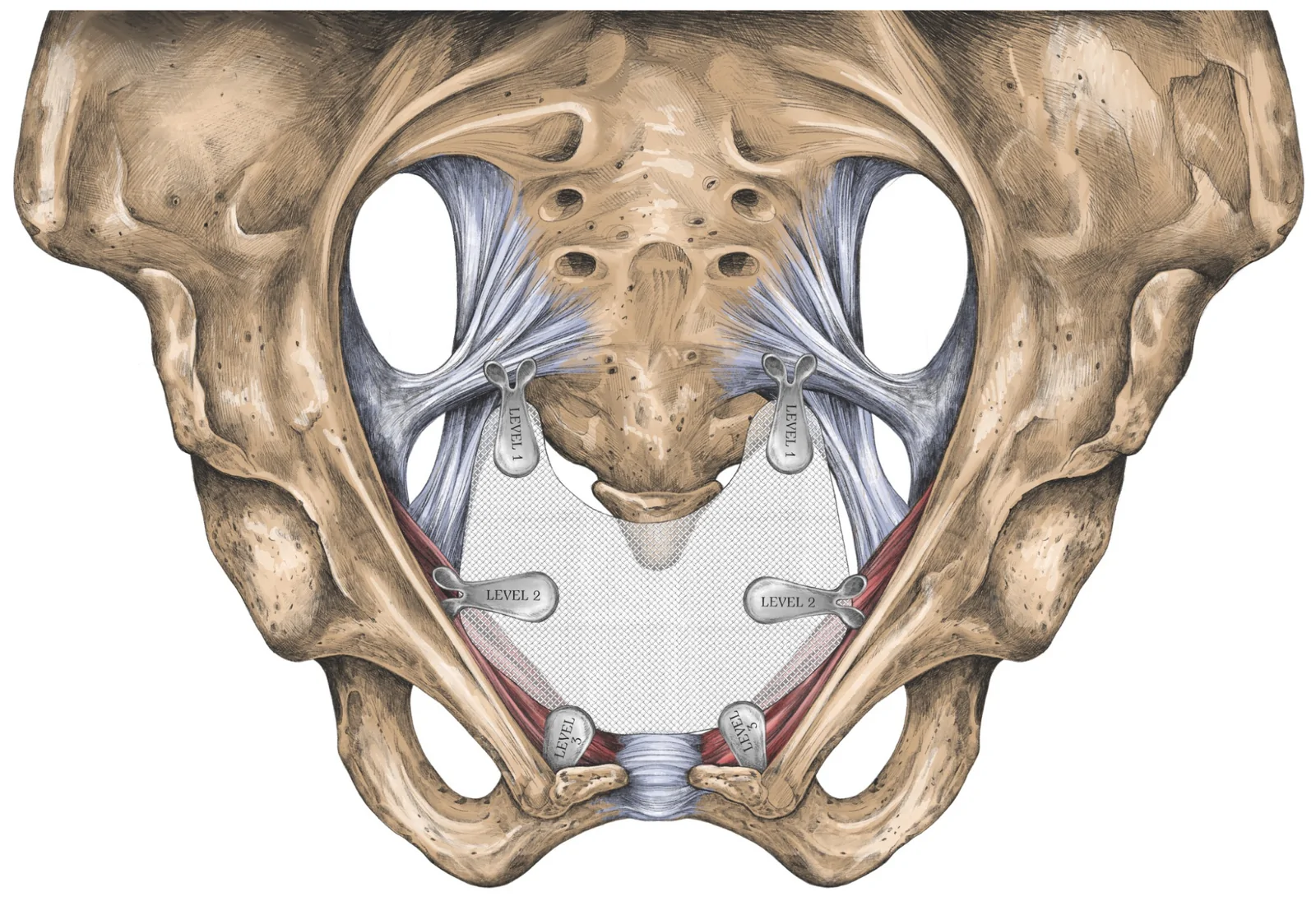
InGYNious anterior mesh and its attachment sites. Illustration prepared by Alicja Marczyk.

**Figure 2:**
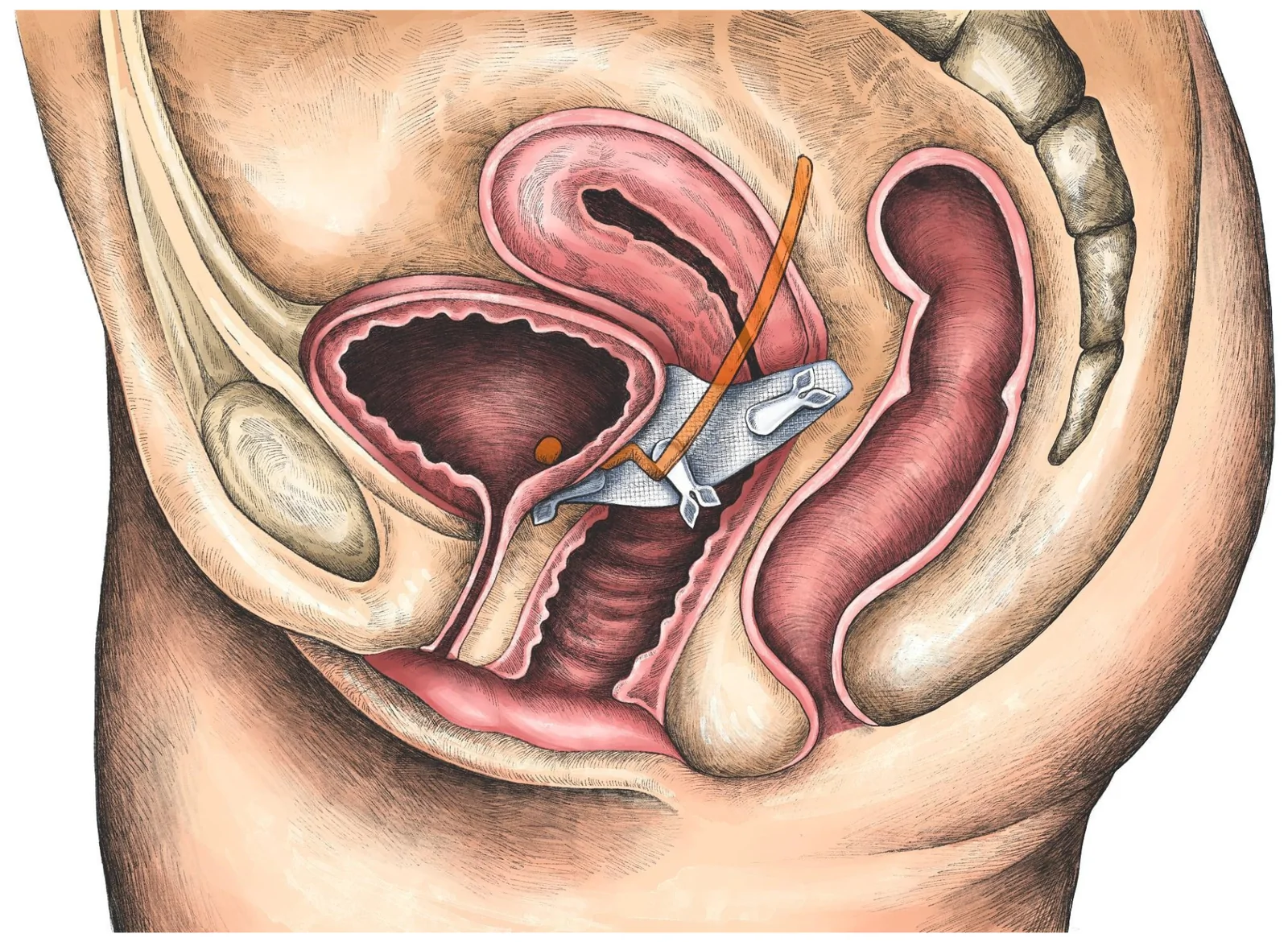
Proposed mechanism of ureteral kinking after InGYNious anterior mesh implantation. Only the left ureter is shown and is represented by the orange line. Illustration prepared by Alicja Marczyk.

**Table 1:** Baseline characteristics of patients with and without urinary tract complications.

Characteristic	No Complications (*n* = 376)	Complications (*n* = 141)	*p*-Value
**Age, median (IQR)**	72 (66–76)	71 (68–75)	0.374
**BMI (kg/m^2^)**			**0.062**
<25	110 (29.3%)	54 (38.3%)	
≥25	266 (70.7%)	87 (61.7%)	
**Smoking**			**0.630**
No	334 (88.8%)	128 (90.8%)	
Yes	42 (11.2%)	13 (9.2%)	
**Vaginal delivery**			**0.670**
0–1	83 (22.1%)	28 (19.9%)	
≥2	293 (77.9%)	113 (80.1%)	
**Caesarean section**			**0.408**
0	354 (94.1%)	136 (96.5%)	
≥1	22 (5.9%)	5 (3.5%)	
**Last menstrual period**			**0.987**
≤10 years	34 (9.0%)	12 (8.5%)	
>10 years	342 (91.0%)	129 (91.5%)	
**Previous hysterectomy**			**0.913**
No	360 (95.7%)	134 (95.0%)	
Yes	16 (4.3%)	7 (5.0%)	
**Previous POP surgery**			**0.728**
No	330 (87.8%)	126 (89.4%)	
Yes	46 (12.2%)	15 (10.6%)	
**SUI**			**0.634**
No	184 (48.9%)	65 (46.1%)	
Yes	192 (51.1%)	76 (53.9%)	
**OAB**			**0.715**
No	285 (76.0%)	110 (78.0%)	
Yes	90 (24.0%)	31 (22.0%)	
**RU**			**0.177**
No	266 (70.7%)	91 (64.5%)	
Yes	110 (29.3%)	50 (35.5%)	
**Obstructed defecation**			**0.932**
No	314 (83.5%)	118 (83.7%)	
Yes	62 (16.5%)	23 (16.3%)	
**Diabetes**			**0.723**
No	277 (73.7%)	101 (71.6%)	
Yes	99 (26.3%)	40 (28.4%)	
**Hypertension**			**0.080**
No	127 (33.8%)	60 (42.6%)	
Yes	249 (66.2%)	81 (57.4%)	
**Dementia**			**0.840**
No	353 (93.9%)	131 (92.9%)	
Yes	23 (6.1%)	10 (7.1%)	
**Depression**			**0.539**
No	359 (95.5%)	137 (97.2%)	
Yes	17 (4.5%)	4 (2.8%)	
**Anemia**			**0.938**
No	364 (96.8%)	137 (97.2%)	
Yes	12 (3.2%)	4 (2.8%)	
**POP-Q anterior stage**			**0.883**
1–2	18 (4.8%)	7 (5.0%)	
3–4	358 (95.2%)	134 (95.0%)	
**POP-Q apical stage**			**0.495**
1–2	19 (5.1%)	10 (7.1%)	
3–4	357 (94.9%)	131 (92.9%)	
**POP-Q posterior stage**			**0.068**
0–2	209 (55.6%)	65 (46.1%)	
3–4	167 (44.4%)	76 (53.9%)	
**Procedure**			**0.043**
Anterior	202 (53.7%)	61 (43.3%)	
Anterior + posterior	174 (46.3%)	80 (56.7%)	
**Hematoma**			**0.793**
No	320 (85.1%)	118 (83.7%)	
Yes	56 (14.9%)	23 (16.3%)	

Data are presented as *n* (%) unless otherwise indicated. Age is presented as median (IQR). *p*-values were calculated using the chi-squared test (categorical variables) and the Mann–Whitney U test (age); values < 0.05 are shown in bold. BMI, body mass index; POP, pelvic organ prolapse; SUI, stress urinary incontinence; OAB, overactive bladder; RU, residual urine; POP-Q, Pelvic Organ Prolapse Quantification; IQR, interquartile range.

**Table 2:** Frequency of urinary tract complications in the study cohort (*n* = 517).

Complication	*n* (%)
Urinary tract infection	14 (2.7%)
Postoperative urinary retention	128 (24.8%)
Conservative or pharmacological management	83 (64.8% of POUR)
Catheterization	45 (35.2% of POUR)
Urinary tract injury	7 (1.4%)
Urethral injury	0
Bladder injury	4 (0.8%)
Ureteral injury or kinking	3 (0.6%)

Percentages for the main complication categories use the total cohort (*n* = 517) as the denominator. Percentages for the two management subcategories of POUR use the 128 patients with POUR as the denominator.

**Table 3:** Univariable logistic regression of risk factors for postoperative urinary retention.

Variable	*p*-Value	OR	95% CI	95% CI
			Lower	Upper
**Age**				
<60 years	Ref.	Ref.	Ref.	Ref.
≥60 years	0.880	0.929	0.358	2.410
**BMI (kg/m^2^)**				
<25	Ref.	Ref.	Ref.	Ref.
≥25	0.106	0.708	0.466	1.076
**Smoking**				
No	Ref.	Ref.	Ref.	Ref.
Yes	0.594	0.832	0.424	1.633
**Vaginal delivery**				
0–1	Ref.	Ref.	Ref.	Ref.
≥2	0.388	1.248	0.754	2.066
**Caesarean section**				
0	Ref.	Ref.	Ref.	Ref.
≥1	0.227	0.513	0.174	1.513
**Last menstrual period**				
≤10 years	Ref.	Ref.	Ref.	Ref.
>10 years	0.889	1.052	0.518	2.137
**Previous hysterectomy**				
No	Ref.	Ref.	Ref.	Ref.
Yes	0.880	1.076	0.415	2.791
**Previous POP surgery**				
No	Ref.	Ref.	Ref.	Ref.
Yes	0.728	0.894	0.474	1.683
**SUI**				
No	Ref.	Ref.	Ref.	Ref.
Yes	0.344	1.214	0.813	1.813
**OAB**				
No	Ref.	Ref.	Ref.	Ref.
Yes	0.807	0.942	0.586	1.516
**RU**				
No	Ref.	Ref.	Ref.	Ref.
Yes	0.104	1.417	0.930	2.159
**Obstructed defecation**				
No	Ref.	Ref.	Ref.	Ref.
Yes	0.990	0.997	0.581	1.709
**Diabetes**				
No	Ref.	Ref.	Ref.	Ref.
Yes	0.410	1.204	0.774	1.873
**Hypertension**				
No	Ref.	Ref.	Ref.	Ref.
Yes	0.156	0.744	0.494	1.120
**Dementia**				
No	Ref.	Ref.	Ref.	Ref.
Yes	0.730	1.150	0.520	2.543
**Depression**				
No	Ref.	Ref.	Ref.	Ref.
Yes	0.538	0.706	0.233	2.138
**Anemia**				
No	Ref.	Ref.	Ref.	Ref.
Yes	0.982	1.013	0.321	3.200
**POP-Q anterior stage**				
1–2	Ref.	Ref.	Ref.	Ref.
3–4	0.701	0.839	0.342	2.056
**POP-Q apical stage**				
1–2	Ref.	Ref.	Ref.	Ref.
3–4	0.216	0.606	0.274	1.340
**POP-Q posterior stage**				
0–2	Ref.	Ref.	Ref.	Ref.
3–4	**0.009**	1.710	1.142	2.559
**Procedure**				
Anterior & apical	Ref.	Ref.	Ref.	Ref.
Anterior & apical + posterior	**0.001**	1.972	1.310	2.967
**Hematoma**				
No	Ref.	Ref.	Ref.	Ref.
Yes	0.490	1.209	0.706	2.071

OR, odds ratio; CI, confidence interval; Ref., reference category. *p*-values < 0.05 are shown in bold. BMI, body mass index; SUI, stress urinary incontinence; OAB, overactive bladder; RU, residual urine; POP, pelvic organ prolapse; POP-Q, Pelvic Organ Prolapse Quantification.

**Table 4:** Multivariable logistic regression of risk factors for postoperative urinary retention.

Variable	*p*-Value	OR	95% CI	95% CI
			Lower	Upper
**Procedure**				
Anterior & apical	Ref.	Ref.	Ref.	Ref.
Anterior & apical + posterior	**0.001**	1.972	1.310	2.967

OR, odds ratio; CI, confidence interval; Ref., reference category. *p*-values < 0.05 are shown in bold.

**Table 5:** Multivariable logistic regression of risk factors for any urinary tract complication.

Variable	*p*-Value	OR	95% CI	95% CI
			Lower	Upper
**Procedure**				
Anterior & apical	Ref.	Ref.	Ref.	Ref.
Anterior & apical + posterior	**0.035**	1.523	1.031	2.249

OR, odds ratio; CI, confidence interval; Ref., reference category. *p*-values < 0.05 are shown in bold.

## Data Availability

The anonymized data supporting the findings of this study are available from the corresponding author upon reasonable request. The data are not publicly available because of privacy and ethical restrictions.

## Abbreviations

AKI	acute kidney injury
ATFP	arcus tendineus fasciae pelvis
BMI	body mass index
CI	confidence interval
CT	computed tomography
eGFR	estimated glomerular filtration rate
EUGA	European Urogynaecological Association
IQR	interquartile range
OAB	overactive bladder
OR	odds ratio
POP	pelvic organ prolapse
POP-Q	Pelvic Organ Prolapse Quantification
POUR	postoperative urinary retention
PVR	post-void residual
SSL	sacrospinous ligament
SUI	stress urinary incontinence
UUI	urgency urinary incontinence
